# Bronchial epithelium repair by Esculentin-1a-derived antimicrobial peptides: involvement of metalloproteinase-9 and interleukin-8, and evaluation of peptides’ immunogenicity

**DOI:** 10.1038/s41598-019-55426-x

**Published:** 2019-12-12

**Authors:** Floriana Cappiello, Danilo Ranieri, Veronica Carnicelli, Bruno Casciaro, Han-Tang Chen, Loretta Ferrera, Y. Peter Di, Maria Luisa Mangoni

**Affiliations:** 1 Laboratory affiliated to Pasteur Italia-Fondazione Cenci Bolognetti, Department of Biochemical Sciences, Sapienza University of Rome, Rome, Italy; 2 Department of Clinical and Molecular Medicine, Sapienza University of Rome, Rome, Italy; 3 Department of Biotechnological and Applied Clinical Sciences, University of L’Aquila, L’Aquila, Italy; 4 Center for Life Nano Science, Istituto Italiano di Tecnologia, Rome, Italy; 5 Department of Environmental and Occupational Health, University of Pittsburgh, Pittsburgh, USA; 6 U.O.C. Genetica Medica, Giannina Gaslini Institute, Genoa, Italy

**Keywords:** Cell biology, Chemical biology

## Abstract

The airway epithelium is seriously damaged upon pulmonary *Pseudomonas aeruginosa* infection, especially in cystic fibrosis (CF) sufferers. Therefore, the discovery of novel anti-infective agents accelerating healing of infected injured tissues is crucial. The antipseudomonal peptides esculentin-1a(1–21)NH_2_ and its diastereomer Esc(1–21)-1c (Esc peptides) hold promise in this respect. In fact, they stimulate airway epithelial wound repair, but no mechanistic insights are available. Here we demonstrated that this process occurs through promotion of cell migration by an indirect activation of epidermal growth factor receptor mediated by metalloproteinases. Furthermore, we showed an increased expression of metalloproteinase 9, at both gene and protein levels, in peptide-treated bronchial epithelial cells with a functional or mutated form of CF transmembrane conductance regulator. In addition, the two peptides counteracted the inhibitory effect of *Pseudomonas* lipopolysaccharide (mimicking an infection condition) on the wound healing activity of the airway epithelium, and they enhanced the production of interleukin-8 from both types of cells. Finally, no immunogenicity was discovered for Esc peptides, suggesting their potential safety for clinical usage. Besides representing a step forward in understanding the molecular mechanism underlying the peptide-induced wound healing activity, these studies have contributed to highlight Esc peptides as valuable therapeutics with multiple functions.

## Introduction

The respiratory epithelium is easily damaged after exposure to microbial pathogens such as *Pseudomonas aeruginosa*^1^. This happens especially in cystic fibrosis (CF) patients due to the establishment of chronic pulmonary bacterial infections^2^. After injury, the lung epithelium starts a wound healing process to re-establish its barrier integrity^3^. An important step during the re-epithelialization event is given by the migration of bronchial epithelial cells in order to reconstitute the epithelial structure and to cover the de-epithelialized area^4–7^. However, tissue repair is hampered in CF patients^8^. Hence, the discovery and selection of novel approaches to treat bacterial lung infections and to further redress the damaged bronchial epithelium is a crucial need. In this context, naturally occurring antimicrobial peptides (AMPs) represent a newsworthy class of therapeutic agents with multiple biological properties, ranging from an antibacterial to an immunomodulatory activity including cure of wounds^9–11^. Recent studies conducted in our laboratory have highlighted that the N-terminal fragment of the frog skin AMP esculentin-1a, i.e. Esc(1–21), GIFSKLAGKKIKNLLISGLKG-NH_2_^12^, and its diastereomer Esc(1–21)-1c bearing two amino acids in the D-configuration (Leu^14^ and Ser^17^) can kill both the planktonic and biofilm life forms of *P. aeruginosa*^13–16^. Furthermore, the two peptides were found to reduce the number of *Pseudomonas* cells internalized into bronchial epithelial cells, expressing either a functional (wt-CFBE cell line) or a defective form of CF transmembrane conductance regulator (CFTR), due to the deletion of phenylalanine at position 508 (F508del-CFBE cell line). This latter is the most common mutation in CF^17^. In addition, both Esc(1–21) and its diastereomer (Esc peptides) were able to advance the healing of a pseudo-wound produced in a monolayer of wt-CFBE and F508del-CFBE, by activation of epidermal growth factor receptor (EGFR), with a higher efficacy for the diastereomer^17^. This function is extremely advantageous, considering that the recovery of an injured infected tissue does not only require elimination of microorganisms but also retrieval of tissue integrity and its barrier function preventing pathogens penetration.

It was previously demonstrated that the wound healing activity of the human AMP LL-37 on epithelial cells occurs through trans-activation of EGFR^18^, mediated by metalloproteinases (MPs), but no information on the type of MPs was provided^18^.

Among MPs, the matrix MMP-9 (92-kDa gelatinase B) is an endopeptidase which is typically activated during tissue injury^19^. It has various roles in growth, development, inflammation and wound healing particularly related to extracellular matrix remodeling and re-epithelialization^20–22^. An enhanced expression and activity of MMP-9 has been identified in many chronic wound types^23,24^, as well as in response to injury, also in the cornea^25^.

In this work, to get insight into the molecular mechanism underlying the Esc peptides-induced closure of a gap produced in a monolayer of wt-CFBE and F508del-CFBE, we initially investigated the effect of the two peptides on the shape of such bronchial cells, especially at the cell’s front edge, along with the contribution of cell proliferation in the re-epithelialization event. Subsequently, in order to know the potential involvement of MPs, we studied the wound healing power of the peptides after treating CFBE with the MP inhibitor GM6001 or the MMP-9 inhibitor I and examined the effect(s) of Esc peptides on MMP-9 expression at both gene and protein levels. In addition, since one of the mechanisms used by host defense peptides to counteract infections consists in an enhanced production of chemokines^26^, secretion of interleukin-8 (IL-8) from CFBE was evaluated. This is because IL-8 is a cytokine which is specifically related to epithelial cells regeneration^27^. Indeed, an increased production of IL-8 was formerly reported to elicit wound reparation in fibroblast layers^28^ and migration process in human epithelial cells^27^. Here, IL-8 level was determined after treating both bronchial cell lines either with the peptides alone or with the peptides’ combination with *P. aeruginosa* lipopolysaccharide (LPS) to mimic a lung bacterial infection condition. Finally, considering the potential usage of Esc peptides as therapeutic agents, their immunogenicity was also evaluated. Remarkably, this is the first report showing the involvement of MMP-9 in the AMPs-induced migration of bronchial epithelial cells, either wt-CFBE or F508del-CFBE, as well as the induction of IL-8 production from Esc peptides-stimulated bronchial cells also in bacterial infection-mimicking conditions. Furthermore, we demonstrated for the first time that Esc peptides are not immunogenic.

## Results

### Effect(s) of Esc peptides on the morphology of CFBE

**Figure 1:**
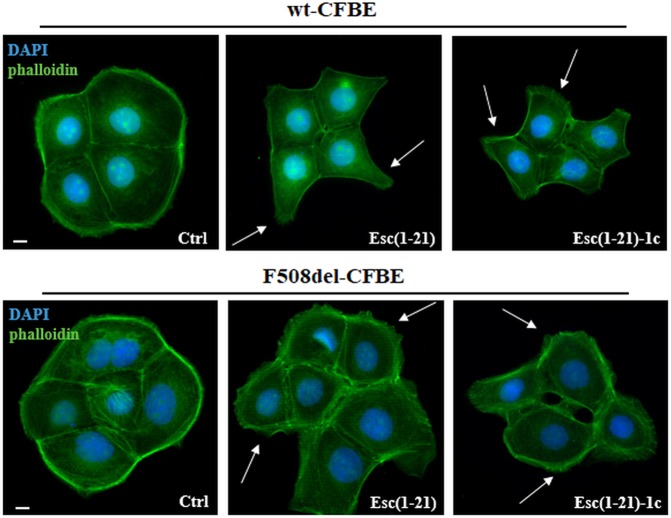
Effects of Esc(1–21) and Esc(1–21)-1c on the shape of CFBE cells. The cells were fixed in formaldehyde and stained with DAPI and phalloidin after 20 h treatment with 10 μM Esc(1–21) or 1 μM Esc(1–21)-1c. The formation of cytoplasmic protrusions in peptide-treated cells are indicated by arrows. Scale bars, 10 μm.

We recently showed that both Esc peptides promoted the restitution of the pseudo-wound produced in wt-CFBE and F508del-CFBE monolayers within 20 h, at an optimal concentration of 10 μM or 1 μM for Esc(1–21) or its diastereomer, respectively^17^. In this current work the effect of Esc peptides on the shape of CFBE cells was investigated by fluorescence microscopy after phalloidin and 4′,6-diamidino-2-phenylindole (DAPI) staining for cytoskeleton detection and nuclei visualization, respectively. Untreated control cells (Ctrl) showed a regular actin cortex and appeared rounded and associated (Fig. 1 left panels). Conversely, cells incubated either with Esc(1–21) or Esc(1–21)-1c (Fig. 1 central and right panels, respectively) appeared stretched, with an altered organization of actin filaments and cytoplasmic protrusions (indicated by arrows). This is consistent with an enhanced cell motility. The different phenotype between the polygonal control cells and the elongated peptide-treated cells was statistically significant (Supplementary Table S1).

**Figure 2:**
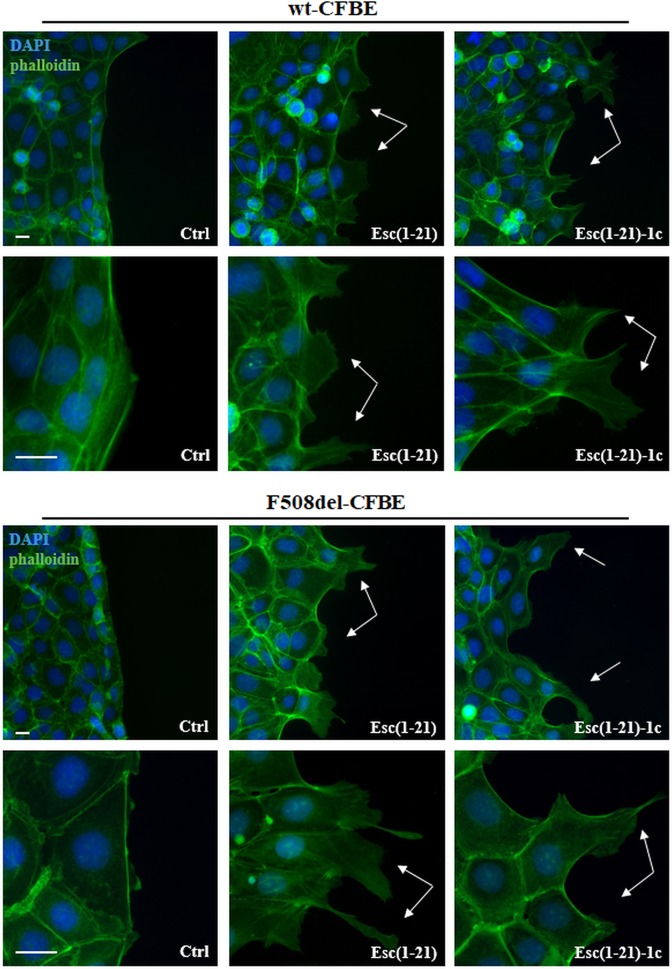
Representative images of the wound edge of CFBE cells, at different magnification, after peptide treatment compared to Ctrl. CFBE cells were seeded in the compartments of silicon chambers placed on glass coverslips. After removal of chambers, samples were treated for 12 h with 10 μM Esc(1–21) or 1 μM Esc(1–21)-1c. Subsequently, cells were fixed and stained for nuclei and cytoskeleton visualization. Unlike untreated Ctrl, each peptide advanced the formation of cytoplasmic protrusions (indicated by the arrows) in migrating cells along the edge of the pseudo-wound produced in both wt-CFBE and F508del-CFBE monolayers. Scale bars, 20 μm.

More importantly, as reported for LL-37 on NCI-H292 cells^29^, such outcome became more evident in both cell lines when analyzed at the edge of the cell monolayer facing the pseudo-wound area and corresponding to the cell migration front. Samples were visualized at a time (12 h) earlier than the complete closure of the gap. As indicated in Fig. 2, we found that stimulation with Esc(1–21) or Esc(1–21)-1c induced a typical migratory phenotype in cells located at the edge of the pseudo-wound with an elongated shape, lamellipodia and ruffles (Fig. 2, arrows). This is better appreciated at a higher magnification for both cell monolayers (Fig. 2, lower panels). On the contrary, no appreciable changes were noted in untreated control samples; in fact, cells remained cobblestone-shaped, tightly associated and they conserved the actin cytoskeleton organized in peripheral cortical bundles (Fig. 2, left panels).

### Role of cell proliferation in the wound healing activity

**Figure 3:**
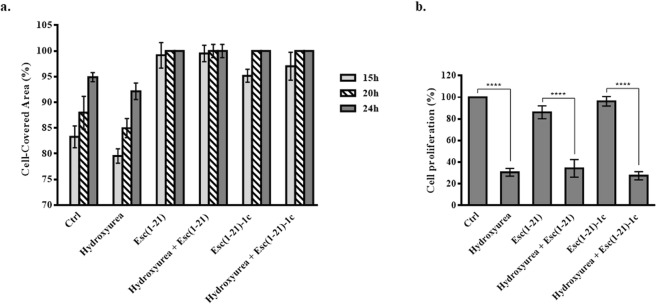
Panel a: Quantitative assessment of cell migration in a pseudo-wound field produced in a monolayer of wt-CFBE cells. Cells were seeded and grown to confluence in each side of silicon chambers. After chamber removal, cells were treated with 250 μM hydroxyurea and 10 μM Esc(1–21) or 1 μM Esc(1–21)-1c. Some samples were incubated with each peptide or hydroxyurea alone, while cells incubated in culture medium were used as control (Ctrl). All experiments were performed four times in triplicates. Data are presented as percentages of cell-covered area at each time point. Percentages of samples treated with the combination hydroxyurea + peptide (with respect to samples treated with the peptide alone) were normalized to those previously obtained when CFBE were treated with each single peptide^17^. In parallel, percentages of samples treated with hydroxyurea alone (with respect to Ctrl) were normalized to those previously obtained for Ctrl samples^17^. All data are expressed as the mean ± standard errors of the mean (SEM). Panel b: Quantitative evaluation of cell proliferation after hydroxyurea treatment. Wt-CFBE cells (2 × 10^4^) were seeded in each well of a microtiter plate. After overnight incubation, cells were treated with 10 μM Esc(1–21), 1 μM Esc(1–21)-1c, 250 μM hydroxyurea or the combination of each peptide with hydroxyurea, for 24 h. After 2 h, cells were pulsed with BrdU. Cell proliferation was normalized to that of cells growing in culture medium (100% cell proliferation; Ctrl) and indicated as percentage. All data are the mean of three independent experiments ± SEM. Significance levels between groups is defined as *P* value of <0.0001 (****).

In order to exclude the contribution of cell proliferation in the peptide-incited restoration of the integrity of CFBE monolayers, the wound healing assay was performed in the presence of the cell proliferation blocker hydroxyurea (Fig. 3a). Since no statistically significant difference was noted between the percentage of cell-covered area in samples treated with hydroxyurea plus each peptide and those treated with the peptide alone at all time intervals, this means that proliferation of wt-CFBE cells is not essential for the re-epithelialization event. Similar results were obtained in the case of F508del-CFBE cells and therefore are not shown.

To ascertain whether the concentration of hydroxyurea used for the wound healing assay was sufficient to affect wt-CFBE cells’ proliferation, quantification of incorporated bromodeoxyuridine (BrdU) into growing cells, was then analyzed (Fig. 3b). The amount of proliferating cells dropped down to ~25% after treatment with hydroxyurea in comparison to control cells (Ctrl). A comparable decrease in cells amount was observed when hydroxyurea was mixed with each Esc peptide (Fig. 3b). Similar data were obtained for F508del-CFBE cells (results not shown). Overall, these findings have contributed to rule out the possibility that the lacking inhibition of wound healing activity by Esc peptides when cells were exposed to the cell proliferation blocker hydroxyurea, was due to an inactive dosage of this latter.

### Involvement of MPs in the mechanism of peptide-induced cell migration

**Figure 4:**
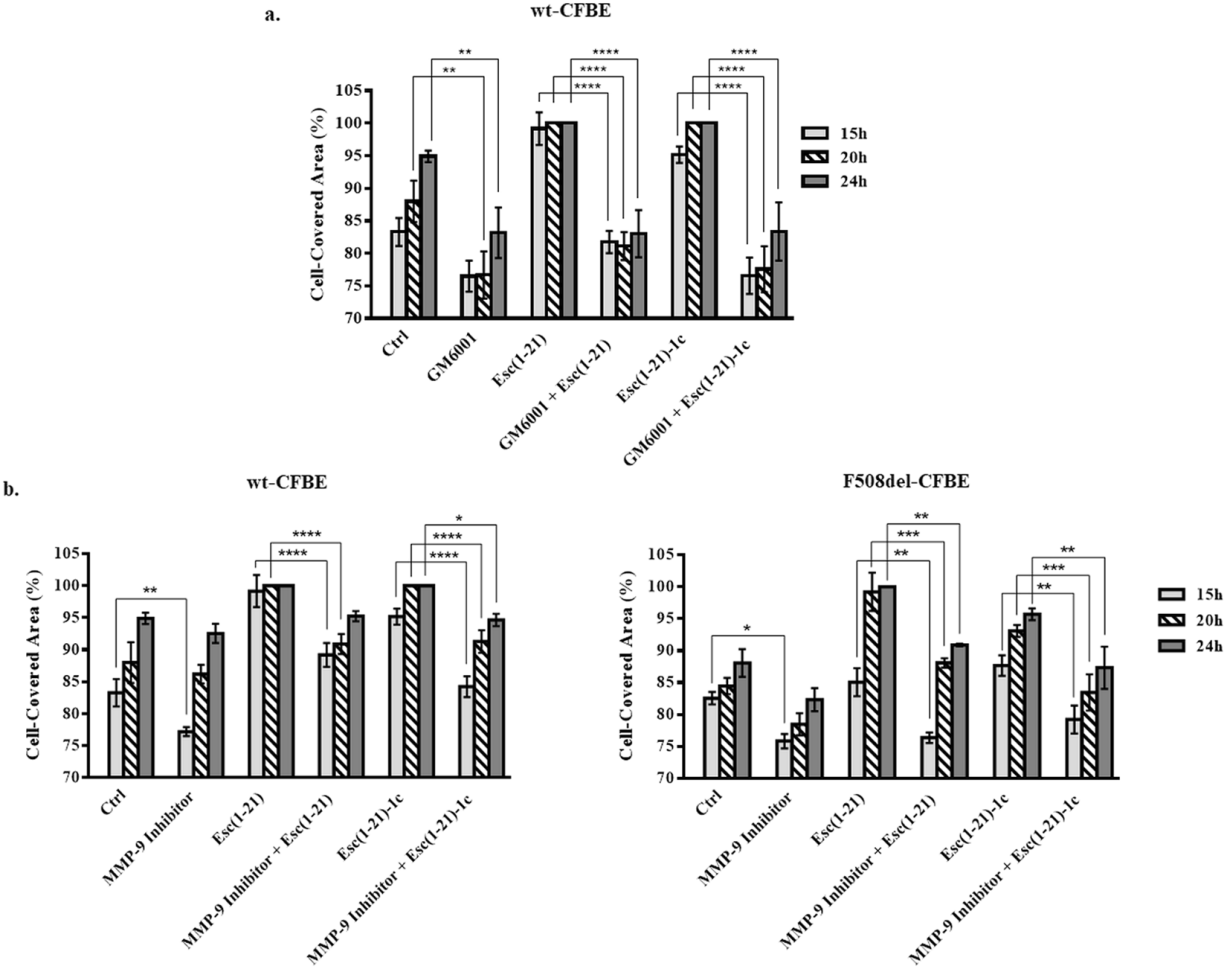
Effect of GM6001 inhibitor (panel a) and MMP-9 inhibitor I (panel b) on the peptide-induced migration of CFBE cells. Before removing the silicone chamber, cells were pre-incubated for 30 min with GM6001 (25 μM) or with MMP-9 inhibitor I (20 nM and 50 nM for wt-CFBE and F508del-CFBE cells, respectively) and subsequently treated with 10 μM Esc(1–21) or 1 μM Esc(1–21)-1c + inhibitor. Some samples were treated with the peptide or inhibitor alone, at the same concentration used in the combination, while untreated cells were used as control (Ctrl). All experiments were repeated four times in triplicates. Cell-covered area was expressed as percentage at all time points. Percentages of samples treated with the combination inhibitor + peptide (with respect to samples treated with the peptide alone) were normalized to those previously obtained when CFBE were treated with each single peptide^17^. In parallel, percentages of samples treated with the inhibitor alone (with respect to Ctrl) were normalized to those previously obtained for Ctrl samples^17^. The results are the mean ± SEM. The levels of statistical significance among groups are *P* values of <0.05 (*), <0.01 (**), <0.001 (***) and <0.0001 (****).

To assess the participation of MPs in the Esc peptides-induced repair of bronchial epithelium, wt-CFBE were pre-incubated with the broad-spectrum MP inhibitor GM6001. As shown in Fig. 4a, treatment of cells with GM6001 prevented the closure of the pseudo-wound field (produced in the cell monolayer), upon peptide treatment as proved by the statistically significant differences in the percentage of cell-covered area at all time intervals (15 h, 20 h and 24 h) with respect to the complete coverage of the gap area when cells were incubated with each single peptide without GM6001. Note that the minor closure of the pseudo-wound in samples treated with GM6001 compared to Ctrl was not due to a cytotoxic effect of the inhibitor (~90% viable cells after 24 h treatment with 25 μM of GM6001) but rather to the inhibition of metalloproteinases activity, which is known to play a crucial role in the re-epithelialization process^30^.

Furthermore, to investigate the involvement of MMP-9 in the wound healing process elicited by Esc peptides, the *in vitro* pseudo-wound healing activity was studied after incubation of wt-CFBE and F508del-CFBE cells with MMP-9 inhibitor I. As reported in Fig. 4b, the re-epithelialization of the wounded field in wt-CFBE was total after 20 h incubation with each Esc peptide. On the contrary, this effect was substantially slower when cells were exposed to the combination of peptide plus MMP-9 inhibitor at almost all time intervals (*P* < 0.0001 after 15 h and 20 h, for Esc(1–21); *P* < 0.0001 after 15 h and 20 h, and *P* < 0.05 after 24 h in the case of the diastereomer). Similarly, in F508del-CFBE cells (Fig. 4b), treatment with each peptide in combination with MMP-9 inhibitor significantly hampered the gap closure at 15 h, 20 h and 24 h compared to what obtained for the peptides alone.

**Figure 5:**
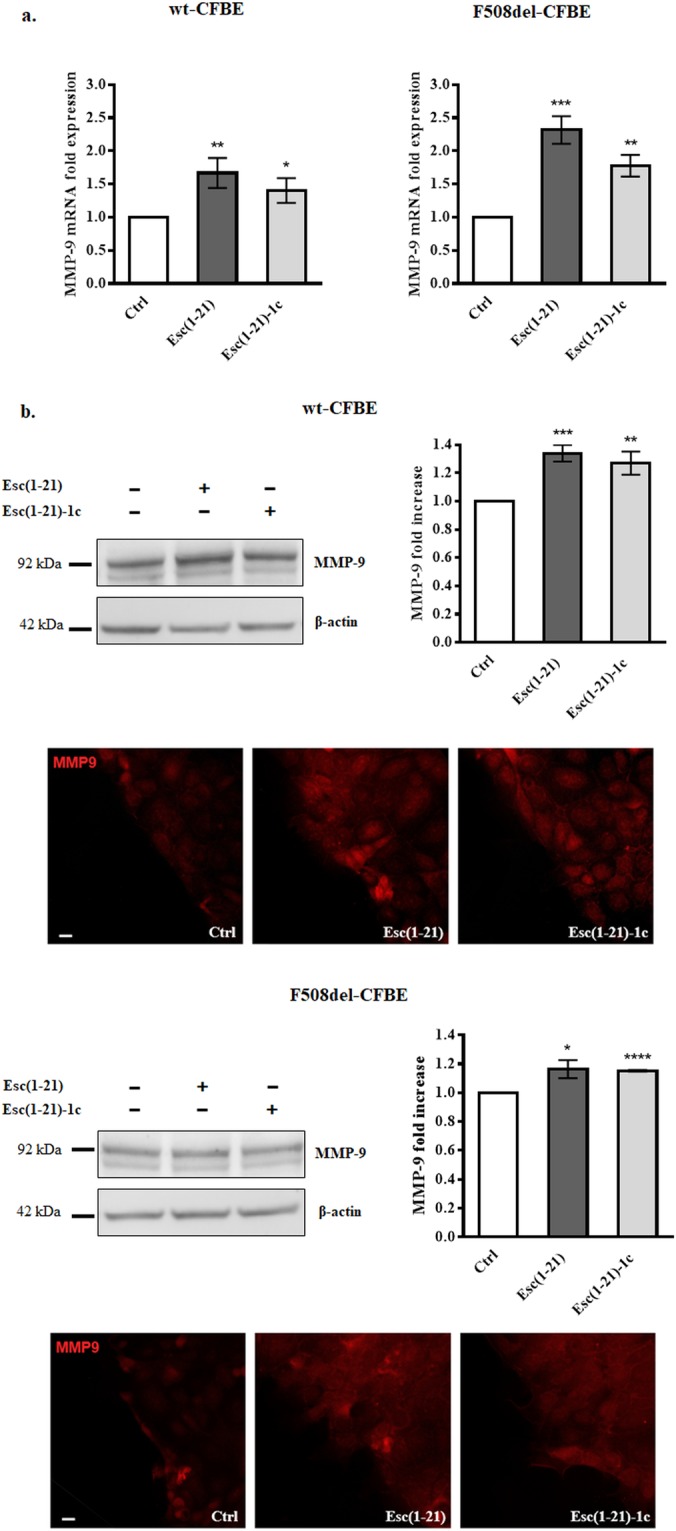
Panel a: Effect of peptides treatment on MMP-9 mRNA expression in wt-CFBE and F508del-CFBE. 3 × 10^5^ CFBE cells were seeded in 35-mm dish plates and treated with Esc(1–21) at 10 μM or Esc(1–21)-1c at 1 μM for 12 h. Afterwards, cells were lysed and the extracted RNA was analyzed by real-time PCR. Results are expressed as fold increase of MMP-9 expression with respect to the untreated Ctrl and indicate the mean value ± SEM of three independent experiments. Significance levels are defined as *P* values of <0.05 (*), <0.01 (**) and <0.001 (***) *versus* the corresponding Ctrl. Panel b: Effect of peptides treatment on MMP-9 protein expression in wt-CFBE and F508del-CFBE. Representative western blots showing the expression of MMP-9 and β-actin in untreated (-) samples or in cells treated with Esc(1–21)/Esc(1–21)-1c, at 10 μM/1 μM, for 12 h (cropped images). Molecular weights of MMP-9 and β-actin are also indicated. Samples showed in the gels derive from the same experiment and were processed in parallel. Full-length images are reported in Supplementary Fig. S1. Histograms indicate the quantitative analysis of MMP-9 measured by densitometry scanning of western blots. Results were normalized and expressed as fold increase with respect to Ctrl and indicate the mean value ± SEM of three independent experiments. Significance levels are defined as *P* values of <0.05 (*), <0.01 (**), <0.001 (***) and <0.0001 (****) *versus* the corresponding untreated Ctrl. Representative images of immunofluorescence to evaluate MMP-9 expression are also reported. Cells were stained with MMP-9 antibody as described in Materials and Methods. Scale bars, 20 μm.

Next, we explored the effect of Esc peptides (at the optimal pseudo-wound healing concentrations) on the production of MMP-9 by bronchial epithelial cells. This was done by studying the expression of MMP-9 at both transcriptional and protein levels at a time point (12 h) earlier than the time intervals used to analyze cell migration upon addition of the peptides. Interestingly, the two AMPs significantly raised the expression of MMP-9 in both cell lines (Fig. 5a).

This finding was also supported by western blotting and immunofluorescence studies on wt-CFBE and F508del-CFBE (Fig. 5b), pointing out a higher production of MMP-9 protein by bronchial cells upon exposure to Esc peptides compared to Ctrl samples.

### Effect of peptides on IL-8 production by bronchial epithelial cells

**Figure 6:**
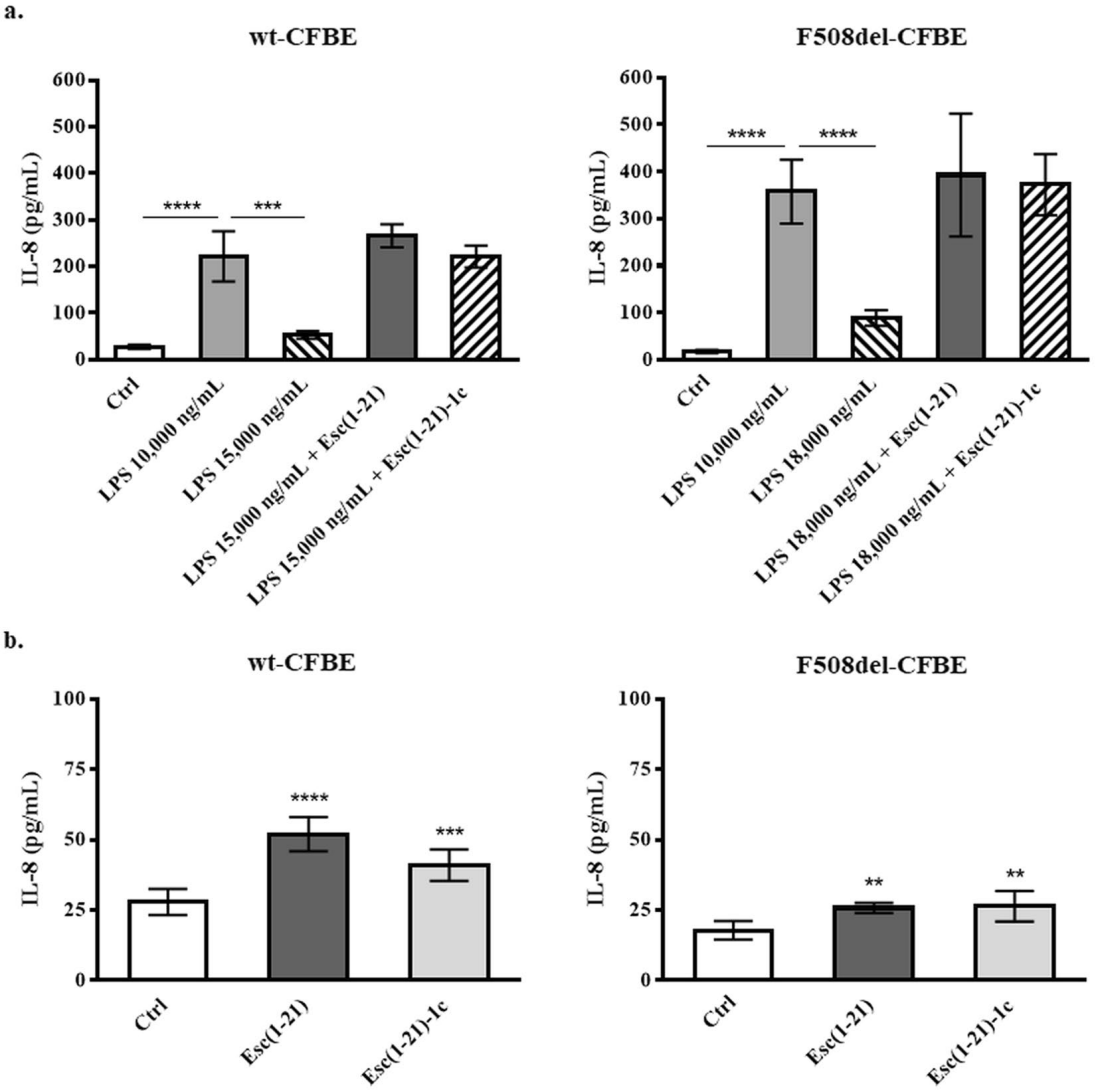
Effect of LPS alone and in combination with Esc(1–21) (4 μM for wt-CFBE and 1 μM for F508del-CFBE) or with 1 μM of Esc(1–21)-1c (panel a) and effect of each Esc peptide alone (panel b) on IL-8 production from wt-CFBE (left panels) and F508del-CFBE cells (right panels), 20 h after treatment. Data are presented as the mean of four independent experiments ± SEM. The level of statistical significance between untreated Ctrl and Esc(1–21)/Esc(1–21)-1c treated cells or between other treatment groups are shown as: **P < 0.01; ***P < 0.001; ****P < 0.0001.

Taking into account the role of IL-8 as a cell migration and chemotaxis factor as well as a wound healing promoter^28,31,32^, the secretion of IL-8 from both wt-CFBE and F508del-CFBE was first evaluated after treatment of cells with *P. aeruginosa* LPS to simulate an infection condition. As reported in Fig. 6a, *P. aeruginosa* LPS at 10,000 ng/mL provoked a substantial production of IL-8, in both cell lines, with the amount of secreted cytokine being 2-fold higher from F508del-CFBE (~400 pg/mL) compared to wt-CFBE (~200 pg/mL). However, at those concentrations of LPS (i.e., ≥15,000 ng/mL) frequently present in the sputum of CF patients with chronic *P. aeruginosa* lung infection^33^, the level of released IL-8 dropped down to 50 or 100 pg/mL for wt-CFBE and F508del-CFBE, respectively (Fig. 6a).

Noteworthy, we recently discovered that pseudo-wound repair in bronchial cell monolayers was impeded when LPS was used at the concentration of 15,000 ng/mL and 18,000 ng/mL for the wt-CFBE and F508del-CFBE^17^, respectively. However, when these concentrations of LPS were mixed with Esc(1–21) or Esc(1–21)-1c, the wound-healing activity was clearly restored^17^. Similarly, also in this case, the combination of LPS at 15,000 or 18,000 ng/mL with each single peptide resets the production of IL-8, up to the levels found with 10,000 ng/mL of LPS (Fig. 6a). As indicated in Fig. 6b and in line with what obtained for LL-37 and human defensins^18,34^, both Esc peptides were able to slightly stimulate the secretion of IL-8 from bronchial epithelial cells (detection levels below 50 pg/mL). Nevertheless, this effect was about 4-fold and 16-fold lower than what detected from wt-CFBE and F508del-CFBE respectively, when the two peptides were administered together with LPS (Fig. 6a).

### Immunogenic studies

**Figure 7:**
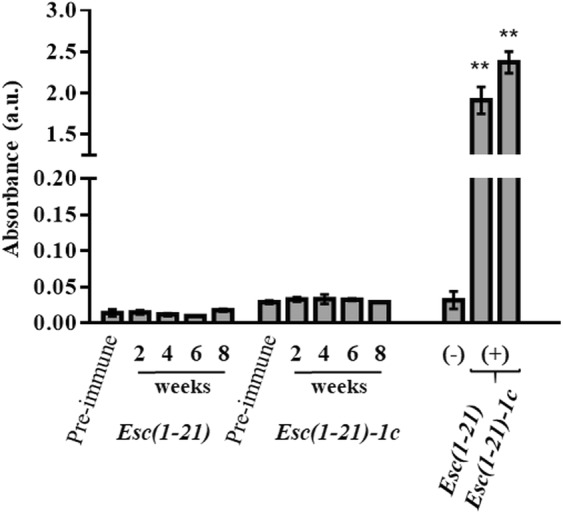
Determination of antibody titer against Esc peptides, by the ELISA assay, in the serum of repeatedly immunized mice at different weeks at which serum was harvested after the original injection. The negative control is represented by undiluted serum from age mice, while the positive controls are given by antibodies against each peptide (diluted 1:6,400). Pre-immune serum was harvested before the first peptide injection. Results represent the mean ± standard deviation (SD) of absorbance. n = 10 mice for each treatment group. *P* < 0.01 (**) was significant for positive controls *versus* all the other groups.

One of the essential requirements for the application of AMPs as new therapeutics is the evaluation of their toxicity and immunogenicity. We previously demonstrated that Esc peptides, especially the diastereomer Esc(1–21)-1c, do not have any noticeable *in vitro* toxic effect against different types of mammalian cells up to 256 μM^14,17^ or an *in vivo* toxicity upon pulmonary administration in mice^35^. Here, the immunogenicity of both peptides was assessed following the standard immunization schedule without adjuvant by subcutaneous (SQ) injection in mice. To increase the possibility of generating immunogenicity, we performed weekly boost with SQ peptide injection for seven consecutive weeks instead of only 2 to 3 boosts during the immunization course. Remarkably, no antibody titer against Esc peptides could be detected in the serum of repeatedly immunized mice (Fig. 7). This is indicated by the invariant absorbance values (obtained by enzyme-linked immunosorbent assay, ELISA) in the serum collected at different weeks after the original peptide injection, compared to the absorbance values of pre-immune serum (harvested before peptide administration to preclude any antigenicity) or negative control (serum from age mice). This demonstrated that both Esc peptides have no immunogenic activities.

## Discussion

In this work, we discovered that treatment of CFBE with both Esc peptides (at a concentration range from 1 to 10 μM) in serum-free medium led to a clear change of the cytoskeleton arrangement, with a cellular shape that is typical of cell motility and that mainly consists in the formation of lamellipodia at the front edge. Furthermore, we found that the peptide-induced mending of the wounded field produced in bronchial cell monolayers was not dependent on cell proliferation. Indeed, treatment of bronchial epithelial cells with hydroxyurea did not bias the coverage pattern of the gap area incited by each individual peptide, suggesting that the re-epithelialization process advanced by Esc peptides is practically driven by cell migration. Differently, a comparable concentration of the human cathelicidin LL-37 (5 μg/mL) stimulated proliferation of human airway epithelial cells^29^, while the airway epithelial wound closure spurred by this AMP required the presence of serum^29^.

Despite previous studies underlined that the healing of a pseudo-wound in bronchial epithelium upon addition of Esc peptides involves activation of EGFR^17^, any further details on the mechanism controlling such event were supplied. Here we have demonstrated the contribution of MPs in the Esc peptides-promoted migration of bronchial epithelial cells, as highlighted by the limited ability of these peptides to restore the entirety of damaged bronchial epithelium, when they were treated with the MPs inhibitor GM6001. More specifically, MMP-9 was found to be implicated, as pointed out by the impaired healing activity of the two Esc peptides, when bronchial cells were incubated with the MMP-9 inhibitor I (Fig. 4). Moreover, as experienced by real-time PCR or western blotting/immunofluorescence analysis, both Esc peptides highly augmented the production of MMP-9, either at transcriptional or protein levels. In the lung, MMP-9 is produced by bronchial epithelium, club cells, alveolar type II cells, smooth muscle, endothelial cells^22^, and its activation is fully involved in epithelial cell migration. In fact, following MMP-9 suppression, airway epithelial cells do not move further^36^. The higher intensity of immunostaining in peptide-treated cell monolayers compared to the untreated ones strongly supported the role of MMP-9 in the peptides-triggered wound-repair of bronchial epithelium expressing either a functional or a defective CFTR.

Another important finding of this study is the secretion of IL-8 from CFBE upon exposure to *Pseudomonas* LPS, the main bacterial pathogen in CF. IL-8 was described as a cytokine produced by the airway epithelium in response to various stimuli including bacterial (*Pseudomonas*) LPS, via Toll-like receptor 4 (TLR4)-EGFR mediated pathway^31,37^. Mechanistically, LPS would bind TLR4 in its monomeric form^38^. However, at dosages higher than the critical micellar concentration (14,000 ng/mL) and that mimic the establishment of a bacterial infection, LPS aggregates^39^. Such large-size particles^39^ would interfere with the binding of LPS to TLR4, thus explaining the reduced amount of secreted IL-8 from CFBE, upon administration of LPS at 15,000 or 18,000 ng/mL, for wt-CFBE and F508del-CFBE, respectively (Fig. 6a). Nevertheless, when LPS was combined with each Esc peptide, a substantial boost of IL-8 secretion was obtained, likely due to dissociation of LPS aggregates into smaller size particles. In support of this, foregoing light scattering analysis underlined that both peptides are able to disrupt LPS micelles^14^. This would re-establish the availability of LPS molecules for an efficient binding to TLR-4 and increased production of IL-8 up to 200 pg/mL or 400 pg/mL (for the wild-type or mutated CFBE respectively).

Noteworthy, concentrations of IL-8 ranging from 100 to 200 pg/mL were reported to accelerate wound healing, with spreading of human fibroblasts^28^. In our preceding work^17^, it was shown that when *Pseudomonas* LPS at 15,000 or 18,000 ng/mL (concentrations hindering re-epithelialization of airway wounds) was mixed with each peptide at the concentration used in this study, this was accompanied by retrieval of the pseudo-wound healing activity. It is known that IL-8 is involved in the first step of airway surface epithelium regeneration characterized by airway epithelial cell adhesion and migration^7^ and that it induces chemotaxis of epidermal cells^32^. In addition, this cytokine was found to enhance re-epithelialization of transplanted meshed human skin^40^.

On the basis of these findings, it can be proposed that one possible mechanism by which Esc peptides are able to recondition the normal architecture of an injured lung tissue in the context of a bacterial infection, hinges on the promotion of IL-8 secretion (and likely MMP-9 production) by bronchial epithelial cells.

**Figure 8:**
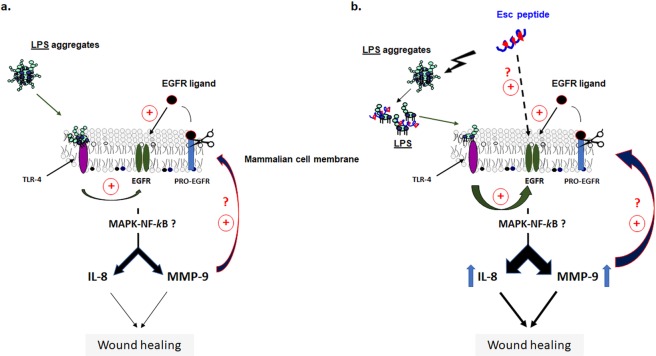
Panel a: Schematic representation of the wound healing activity in airway epithelium by LPS-mediated activation of EGFR to simulate an infection condition. Activation of EGFR leads to IL-8 and MMP-9 production (likely through MAPK signaling pathway) which are involved in the wound healing process. MMP-9 would also contribute to cleave EGFR-proligands, thus leading to transactivation of EGFR. Panel b: The same representation showing the plausible mechanism of enhanced wound healing process after Esc peptides treatment. By disrupting LPS aggregates into smaller size particles, Esc peptides would allow LPS monomers to activate EGFR, *via* LPS-TLR4-mediated pathway. In addition, peptides treatment provokes an increased expression of IL-8 and MMP-9.

As stated above, the wound healing activity of Esc peptides in CFBE monolayers implies the involvement of MMP-9 by presumably favoring the disassembly of the extracellular matrix, and the cleavage of pro-EGFR ligands^41^. In fact, several matrix MPs can cleave cell surface bound pro-EGFR ligands^41–45^. Free EGFR ligands would then bind and activate EGFR, triggering the NF-kB-mediated pathway, possibly through upstream MAPK signaling network to finally raise MMP-9 transcripts and proteins as well as the production of IL-8 (See schematic representation in Fig. 8).

In summary, this is the first report showing that the Esc peptides-induced wound repair of bronchial epithelium expressing either a functional CFTR or its common mutated form in CF: (i) relies on the migration of bronchial cells rather than on their proliferation; and that (ii) it engages the participation of MMP-9. Note that so far no indication on the type of MP involved in the mechanism of pseudo-wound healing induced by AMPs at the airway epithelium has been described. Moreover, our results have indicated the ability of these peptides to counteract the inhibitory effect of pulmonary *Pseudomonas* infection (characterized by high concentrations of LPS, especially in CF) on the migration (wound healing) of CFBE by enhancing IL-8 secretion from these cells, with a higher effectiveness for the diastereomer. This highlights IL-8 as a determinant molecule in the process of re-epithelialization.

Finally, only a few studies have been carried out so far to investigate the immunogenicity of short peptides. Here, for the first time we have also demonstrated that both Esc peptides are not immunogenic in animals, thus encouraging and ensuring their development for clinical usage.

## Materials and Methods

### Materials

Synthetic Esc(1–21) and its diastereomer Esc(1–21)-1c were purchased from Biomatik (Wilmington, USA). Minimum essential medium (MEM), glutamine, heat inactivated fetal bovine serum (FBS), trypsin-EDTA and penicillin-streptomycin were from Euroclone (Milan, Italy); puromycin, hydroxyurea, Triton X-100, DAPI, Mowiol 4–88, phalloidin-fluorescein isothiocyanate, LPS from *P. aeruginosa* serotype 10 (purified by phenol extraction), bovine serum albumin (BSA), anti-β-actin monoclonal antibody were purchased from Sigma-Aldrich (St. Luis, MO). GM6001 inhibitor, MMP-9 Inhibitor I and BrdU cell proliferation assay kit were from Millipore Merck (Merck, Milan, Italy). Human IL-8 Standard ELISA Kit was purchased from Peprotech (Rocky Hill, NJ, USA). Mouse monoclonal anti-MMP9 (2C3): sc-21733 was purchased from Santa Cruz Biotechnology; goat anti-mouse IgG-Texas Red from Jackson Immunoresearch Laboratories, West Grove, PA. Quick-RNA MiniPrep was purchased from Zymo Research, Irvine, CA, USA. iScript cDNA synthesis kit, iQ SYBR Green Supermix, non-fat dry milk and Tween-20 were from Bio-Rad Laboratories, Hercules, CA, USA. Oligonucleotide primers were purchased from Invitrogen (Carlsbad, CA). All other chemicals were reagent grade.

### Cell cultures

According to what described in^17^, the immortalized human bronchial epithelial cells obtained from a CF patient (CFBE41o-) were transduced with a lentiviral vector for a stable expression of the functional CFTR (wt-CFBE) or F508del CFTR (F508del-CFBE)^46^. Both CFBE cell lines were cultured at 37 °C and 5% CO_2_ in 75-cm^2^ flasks containing MEM supplemented with 2 mM glutamine (MEMg), 10% FBS, antibiotics (0.1 mg/mL of streptomycin and penicillin) and puromycin (0.5 μg/mL or 2 μg/mL for wt-CFBE or F508del-CFBE, respectively).

### Pseudo-wound healing assay

The migration of CFBE cells was evaluated by an alternative method to the classic scratch assay, as described in^47^. Briefly, it was based on the usage of Ibidi culture inserts^48^, properly placed into each well of a 12-well plate^17^. About 35,000 cells, suspended in MEMg plus 10% FBS, were seeded in each compartment of the inserts. Then, cells were incubated at 37 °C and 5% CO_2_ for approximately 24 h to reach confluence. Afterwards, in order to create a cell-free area (pseudo-wound) of approximately 500 μm in the cell monolayer, the inserts were removed and the cell proliferation blocker hydroxyurea^49^, suspended in MEMg at a concentration of 250 μM, was added to each well alone or in combination with 10 μM Esc(1–21) or 1 μM Esc(1–21)-1c. The cells were allowed to migrate and samples were visualized at different time intervals under an inverted microscope (Olympus CKX41) at 4 × magnification and photographed with a Color View II digital camera. The percentage of cell-covered area was calculated by the WIMASIS Image Analysis. Pseudo-wound healing assays were also conducted by treating CFBE cells with a broad spectrum MPs inhibitor, GM6001^50,51^, or with MMP-9 Inhibitor I^52^, to assess the contribution of MPs activity in the peptide-induced cell migration. Samples treated with each single peptide were also included for comparison.

### Cell proliferation studies

Wt-CFBE cells (2 × 10^4^), suspended in MEMg supplemented with 2% FBS, were seeded in each well of a 96-well plate and incubated overnight at 37 °C and 5% CO_2_ atmosphere. Afterwards, the medium was removed and 100 μL of fresh MEMg containing each Esc peptide, hydroxyurea or each peptide in combination with hydroxyurea were added for 24 h. Two hours after the start of the treatment, cells were incubated with BrdU, which is incorporated into newly synthesized DNA of proliferating cells. Cell proliferation, expressed as a percentage, was analyzed by a BrdU cell proliferation assay kit and normalized to that of Ctrl samples (cells growing in MEMg, 100% cell proliferation).

### Fluorescence microscopy and immunofluorescence

About 1.5 × 10^5^ CFBE cells in MEMg, supplemented with 10% FBS, were seeded on 0.13- to 0.17-mm-thick coverslips properly placed into 35-mm dish plates and incubated overnight at 37 °C and 5% CO_2_. Afterwards, CFBE cells were treated with each Esc peptide in MEMg for 20 h; fixed with 4% formaldehyde for about 15 min and permeabilized with 0.1% Triton X-100 in phosphate-buffered saline (PBS) for 5 min, at room temperature. Subsequently, CFBE cells were stained with phalloidin-fluorescein isothiocyanate (40 μM in PBS) for 20 min and with DAPI (1 μg/mL) for 5 min at room temperature to visualize the cytoskeleton and the nuclei, respectively. The coverslips were mounted on slides using Mowiol. Images were obtained by conventional fluorescence with an ApoTome System (Zeiss) connected with an Axiovert 200 inverted microscope (Zeiss) and image analysis was performed by the Axiovision software (Zeiss). Quantitative analysis of cells morphological changes was carried out after 20 h of peptide treatment. Cells were distinct for their migratory phenotype into two subpopulations: elongated and polygonal cells. A cell count of ten photomicrographs from three different experiments was performed (~200 cells). For fluorescence microscopy analysis on cell monolayers and MMP-9 localization, about 3.5 × 10^4^ CFBE cells suspended in MEMg supplemented with 10% FBS, were seeded in each compartment of the culture insert (mentioned above), placed on 0.13- to 0.17-mm-thick coverslips which were put into 35-mm dish plates. After approximately 24 h incubation at 37 °C and 5% CO_2_, the inserts were removed to create a pseudo-wound and each Esc peptide was added in MEMg for 12 h at the indicated concentration. Cell monolayers were fixed and permeabilized as described above. For MMP-9 detection, samples were incubated for 1 h at 25 °C with the mouse monoclonal anti-MMP-9. The primary antibody was visualized using goat anti-mouse IgG-Texas Red (1:200 in PBS) for 30 min at 25 °C. Nuclei were stained as described above. The coverslips were finally mounted as indicated above.

### Primers

The oligonucleotide primers used to target genes and the housekeeping gene, chosen by the online tool Primer- BLAST^53^, were the following: for MMP-9 target gene, 5′-CGCGCTGGGCTTAGATCATT -3′ (sense), 5′-GGGCGAGGACCATAGAGGT-3′ (anti-sense); for the 18 S rRNA housekeeping gene, 5′-AACCAACCCGGTCAGCCCCT-3′ (sense), 5′-TTCGAATGGGTCGTCGCCGC-3′ (antisense). For each primer pair, no-reverse-transcriptase control (RT negative) and no-template control assays were performed, and negligible signals were produced.

### RNA extraction and cDNA synthesis

About 3 × 10^5^ CFBE cells in MEMg supplemented with 10% FBS were seeded in 35-mm dish plates and incubated at 37 °C and 5% CO_2_. The next day, the medium was replaced with fresh MEMg containing Esc(1–21) at 10 μM or Esc(1–21)-1c at 1 μM. After 12 h of treatment, total RNA was extracted using the Quick-RNA MiniPrep. For each sample, 1 μg of RNA was used to reverse transcription using iScript cDNA synthesis kit with thermal cycling programme as follows: 25 °C for 5 min, followed by 46 °C for 20 min and 95 °C for 1 min, as reported^54^.

### PCR amplification and real-time quantitation

The iCycler Real-Time Detection System (iQ5 Bio-Rad) was employed to carry out real-time PCR. By using iQ SYBR Green Supermix, the reaction was performed in 96-well plate; forward and reverse primers for each gene and 14 ng of diluted template cDNA were added to a final reaction volume of 15 μL. All assays were run three times and included a negative control. The thermal cycling program was performed as described^55^. The iCycler IQ optical system software version 3.0a (Bio-Rad Laboratories) was used for real-time quantitation according to the manufacturer’s manual.

### Western blot analysis

Cells were lysed, and total protein was resolved by sodium dodecyl sulphate-polyacrylamide gel electrophoresis (SDS-PAGE) and transferred to reinforced nitrocellulose according to^56^. The membranes were blocked with 5% non-fat dry milk in PBS/0.1% Tween-20 or with 3% BSA in PBS/0.1% Tween-20, and incubated with anti-MMP-9 monoclonal antibodies^57^ followed by enhanced chemiluminescence detection (ECL, Amersham, Alington Heights, IL). The membranes were rehydrated in PBS/Tween-20, stripped with 100 mM mercaptoethanol and 2% SDS for 30 min at 55 °C and probed again with anti-β-actin monoclonal antibody to estimate the protein equal loading. Quantity One program (Bio-Rad) was used for densitometric analysis.

### IL-8 detection by bronchial epithelial cells

CFBE cells (1 × 10^5^) suspended in MEMg supplemented with 10% FBS were seeded in each well of a 24 well plate and incubated for approximately 24 h at 37 °C and 5% CO_2_. Afterwards, the cells were treated with MEMg containing peptide, LPS or their combination, at the indicated concentrations, for 20 h. At the end of treatment, the supernatants were collected, and IL-8 concentration was evaluated by ELISA, according to the manufacturer’s protocol. Cells stimulated with LPS alone and untreated cells served as controls.

### Immunogenicity studies

Wild-type CD-1 male and female mice were purchased from Charles River (Wilmington, MA). All procedures and experiments were conducted according to animal protocol approved by the University of Pittsburgh Institutional Animal Care and Use Committee (IACUC) and performed in accordance with National Institutes of Health guidelines and regulations. After one week acclimation, mice were immunized SQ with 0.1 mL of the Esc peptides (1 mg/mL), once per week for 7 weeks. Serum samples from each mouse were collected weekly before each subsequent injection through drawing blood from tail vein. Pre-immune control of serum samples was obtained by drawing blood from naïve mice before the first peptide injection. Final bleed of blood samples was collected by cardiac puncture at one week after the last injection. To analyze the presence of antibodies against each peptide, all collected serum samples at different time intervals from each mouse were analyzed by an indirect ELISA as previously described^58,59^.

Briefly, serum samples containing antibodies were incubated with pre-coated Esc peptide antigen (1 μg/well), subsequently incubated with anti-mouse-IgG antibody conjugated with horseradish peroxidase, and detected with specific substrate. Rabbit polyclonal antibodies against Esc peptides were used as positive controls^60^. Absorbance measurements of samples were carried out. The absorbance of no peptide sample control (blank) was subtracted from the absorbance values of the corresponding samples.

### Statistical analyses

Quantitative data derived from independent experiments were expressed as the mean ± SEM or SD. Statistical significance was determined using two-way analysis of variance (ANOVA) or Student’s t test, with PRISM software (GraphPad, San Diego, CA). *P* values of <0.05 were assumed to be statistically significant. The levels of statistical significance are indicated in the legend to figures.

## Supplementary information

Supplementary Information
