# Supplementary material for: Bronchial epithelium repair by Esculentin-1a-derived antimicrobial peptides: involvement of metalloproteinase-9 and interleukin-8, and evaluation of peptides’ immunogenicity
Source: Sci Rep. 2019 Dec 12;9:18988. doi: 10.1038/s41598-019-55426-x (PMC6908641; doi:10.1038/s41598-019-55426-x)
Supplement: Supplementary file 1 — Supplementary Information [file 41598_2019_55426_MOESM1_ESM.pdf]

SUPPLEMENTARY INFORMATION

Bronchial epithelium repair by Esculentin-1a-derived antimicrobial peptides: involvement of metalloproteinase-9 and interleukin-8, and evaluation of peptides' immunogenicity

Floriana Cappiello[1], Danilo Ranieri[2], Veronica Carnicelli[3], Bruno Casciaro[4], Han-Tang Chen[5], Loretta Ferrera[6], Y. Peter Di[5], Maria Luisa Mangoni[1*]

[1] Laboratory affiliated to Pasteur Italia-Fondazione Cenci Bolognetti, Department of Biochemical Sciences, Sapienza University of Rome, Italy; [2] Department of Clinical and Molecular Medicine, Sapienza University of Rome, Rome, Italy; [3] Department of Biotechnological and Applied Clinical Sciences, University of L'Aquila, L'Aquila, Italy; [4] Center for Life Nano Science, Istituto Italiano di Tecnologia, Rome, Italy; [5] Department of Environmental and Occupational Health, University of Pittsburgh, Pittsburgh, USA; [6] U.O.C. Genetica Medica, Giannina Gaslini Institute, Genoa, Italy.

*To whom correspondence should be sent:

Maria Luisa Mangoni
Department of Biochemical Sciences,
Sapienza University of Rome
Via degli Apuli, 9;00185, Rome
Tel: +39 06 49917693. Email: marialuisa.mangoni@uniroma1.it
ORCID: 0000-0002-5991-5868

Supplementary Table S1. Quantitative analysis of cell morphological changes was carried out after 20 h treatment of wt-CFBE and F508del-CFBE with 10 μM and 1 μM of Esc(1-21) and Esc(1-21)-1c, respectively, compared to the untreated Ctrl samples. Cells with cytoplasmic protrusions were counted from 10 different fields randomly taken from three different experiments (~ 200 total cells). Results are expressed as percentages mean ± SEM of elongated cells with respect to the total number.

**Percentages of elongated cells**			
**Cell type**	**Ctrl**	**Esc(1-21)-treated cells**	**Esc(1-21)-1c-treated cells**
wt-CFBE	12.14 ± 1.64	60.59 ± 1.73	71.34 ± 4.40
F508del-CFBE	15.05 ± 1.90	86.04 ± 1.50	80.38 ± 1.92

Significance levels between peptide-treated samples versus Ctrl is defined as *P* value of <0.0001 (****).

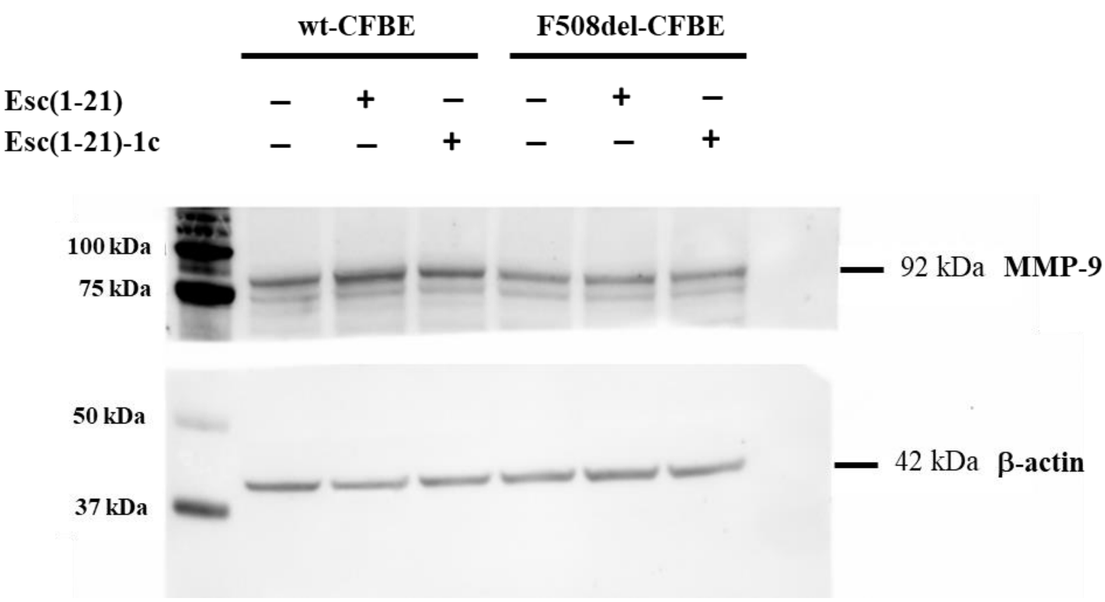

Supplementary Figure S1

Western blots showing the expression of MMP-9 and β–actin in untreated (-) samples or in cells treated with Esc(1-21)/Esc(1-21)-1c, at 10 µM/1 µM, for 12 h. Molecular weights of MMP-9 and β-actin are also indicated. Samples showed in the gels derive from the same experiment and were processed in parallel. **This represents full-length images of cropped scans of Figure 5**
